# Interconnected MXene/Graphene Network Constructed by Soft Template for Multi-Performance Improvement of Polymer Composites

**DOI:** 10.1007/s40820-022-00877-7

**Published:** 2022-06-14

**Authors:** Liyuan Jin, Wenjing Cao, Pei Wang, Na Song, Peng Ding

**Affiliations:** Research Center of Nanoscience and Nanotechnology, College of Sciences, Shanghai University, 99 Shangda Road, Shanghai, 200444 People’s Republic of China

**Keywords:** Structural design, MXene/graphene network, Soft-template, Thermal conductivity, Electromagnetic interference shielding

## Abstract

The multi-functionalization of polymer composites refers to the ability to connect multiple properties through simple structural design and simultaneously achieve multi-performance optimization. The large-scale design and mass production to realize the reasonable structure design of multifunctional polymer composites are urgently remaining challenges. Herein, the multifunctional MXene/graphene/polymer composites with three-dimensional thermally and electrically conductive network structures are fabricated via the utilization of the microstructure of the soft template, and a facile dispersion dip-coating approach. As a result, the polymer composites have a multi-performance improvement. At the MXene and graphene content of 18.7 wt%, the superior through-plane thermal conductivity of polymer composite is 2.44 W m^−1^ K^−1^, which is 1118% higher than that of the polymer matrix. The electromagnetic interference (EMI) shielding effectiveness of the sample reaches 43.3 dB in the range of X-band. And the mechanical property of the sample has advanced 4 times compared with the polymer matrix. The excellent EMI shielding and thermal management performance, along with the effortless and easy-to-scalable producing techniques, imply promising perspectives of the polymer composites in the next-generation smart electronic devices.

**Supplementary Information:**

The online version contains supplementary material available at 10.1007/s40820-022-00877-7.

## Highlights

- Interconnected MXene/graphene networks are constructed by soft template through a facile dispersion dip-coating approach for multi-performance improvement of polymer composites.
- The superior through-plane thermal conductivity, electromagnetic interference shielding, and mechanical property performance of polymer composites are achieved.
- The polymer composites obtained improve phase change and thermomechanical properties, which provided the possibility for the application in variable temperature environments.

**Supplementary Information:**

The online version contains supplementary material available at 10.1007/s40820-022-00877-7.

## Graphical abstract

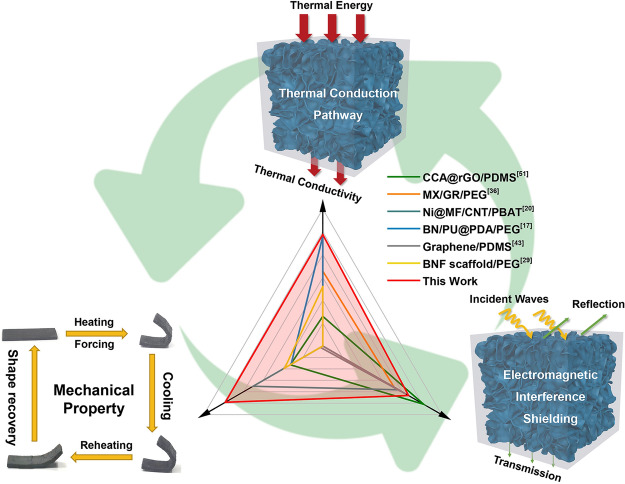

## Introduction

The evolution of miniaturization and integration of electronic devices affects the progress of polymer composites towards the direction of multifunctional integration. The heat accumulation and the released electromagnetic radiation of electronic devices put forward higher requirements of multi-functionalization. Thermal management is the foundation, on which excess heat will not only affect the user experience but also affect the service life. Electromagnetic shielding performance is also tightly correlated to thermal management. When the electromagnetic wave passes through the composite, the electromagnetic wave will be reflected and absorbed by the conductive composition, and then further converted into heat energy [1, 2]. The constant generation of heat affects the polymeric composite, causing a phase change process. Mechanical properties are the basis of applicability. Vibration and deformation of composites will generate heat due to molecular chain movement. Composites need to have spontaneous thermal management properties in practical applications due to the generation and dissipation of heat. Therefore, the realization of multi-functionalization requires that composites are simultaneously improved in thermal management, electromagnetic shielding, phase change properties, and mechanical properties. The construction of a continuous interconnected network has been proved to be the most effective strategy for thermally managed [3, 4] and electromagnetic shielding [5, 6] composites.

Polymer sponges are a kind of continuous natural three-dimensional (3D) soft templates, which can effectively form continuous filler interconnected networks through simple dipping or coating [7–9]. Long-range continuous electrical [10–12] and thermal network [13–15] is an essential structural feature for the formation of multifunctional polymer composites with high performance. The 3D soft templates represented by polyurethane (PU) sponge [11, 16–19], melamine foam [10, 20–22], and polyimide foam [23, 24], combined with functional fillers, can easily obtain the continuous interconnected structure. Compared with the aerogel formed by the hydrogen bonding interaction [25, 26], the network is more supportive in the polymer matrix [16, 27]. The adoption of soft templates to form a 3D interconnected network has been widely studied. Ma et al. [10] used a melamine sponge (MS) to construct a 3D porous MXene@MS composite sponge with a capsule structure. After covering with MXene suspension of 4 mg mL^−1^ for 4 dip-coating cycles, the MXene@MS composite sponge exhibited excellent electromagnetic interference (EMI) shielding effectiveness (SE) of 53.16 dB. Xue et al. [20] fabricated Ni@MF/CNT/PBAT composites with a stepped asymmetric structure by a simple solution encapsulation method. The composites exhibited directional electromagnetic shielding efficiency. When Ni@MF is the incident surface of electromagnetic waves, the average *SE*_T_ of Ni@MF-5/CNT-75/PBAT is 38.3 dB. When CNT is the incident surface of electromagnetic waves, the average *SE*_T_ of the same composite is 29.5 dB, with an 8.8 dB difference. Jiang et al. [17] coated boron nitride nanosheets on the PU sponges to construct a 3D interconnected network structure. After vacuum infiltration of the polymer matrix, the manufactured PBPP composites achieved a high thermal conductivity of 2.4 W m^−1^ K^−1^ at a loading of 17.5 wt%. The above-mentioned application certificates that the soft template method has potential in the preparation of multifunctional composites.

In this work, the 3D thermal and electrical conduction interconnected network was prepared by coating the MXene and graphene on a PU soft template, and then the MXene/graphene/PU composite sponge was encapsulated in molten polyethylene glycol (PEG). The polymer composites obtained superior multi-performance improvement. At the filler content of 18.7 wt%, the through-plane thermal conductivity of polymer composite is 2.44 W m^−1^ K^−1^, which is 1118% superior than that of the pure polymer matrix. The electromagnetic SE of the sample raises to 43.3 dB. The mechanical property of the sample has progressed 4 times. Compared with other methods to prepare 3D interconnected structures, the utilization of the soft template is more convenient, efficient, and has the advantage of commercial production. The prepared polymer composites effectively combine multifunctional performance and have potential application in the next-generation smart electronic devices.

## Experimental Section

### Materials

PU sponges (thickness = 0.5 cm) were purchased from Yongxin Sponge Factory (Zhejiang, China). Nanofibrillated cellulose (NFC) was acquired by Guilin Qihong Technology (1 wt%). Ti_3_AlC_2_ MAX phase (200 mesh) and Graphene (KNG-C162, 98.8%) were purchased from 11 Technology Co., Ltd. (Jilin, China) and Knano Graphene Technology Co., Ltd. (Xiamen, China), respectively. Lithium fluoride (CP), hydrochloric acid (AR), and polyethylene glycol (PEG, Mn = 6000) were purchased from Sinopharm Chemical Reagent Co., Ltd. (Shanghai, China). Dopamine (DA) and 2-Amino-2-(hydroxymethyl)-1,3-propanediol (tris) hydrochloride were purchased from Sigma-Aldrich. All the chemicals were used without any further purification.

### Preparation of NFC/MXene/Graphene Solution

40 mL of 9 M HCl and 2.0 g of LiF powder were added in a 100 mL PTFE beaker to mix the solution. 2.0 g of MAX phase was tardily added to the mixture under an ice bath, followed by further stirring at 35 ℃ for 24 h. Several cycles of diluting with deionized water were repeated by centrifugation (3500 rpm, 10 min for each cycle) until the pH > 5, followed by centrifugation at 3500 rpm for 60 min to acquire the products [28]. 1 mL NFC was adept to deionized water (3 mL) and stirred evenly to configure NFC aqueous dispersion. Graphene and MXene (1:1) with different concentrations (25, 50, 75, 100 mg mL^−1^) were ultrasonicated and stirred alternately for 4 times and 15 min each time, and followed by stirring for 1 h. The uniform NFC/MXene/Graphene solution was obtained. The morphology of MXene and graphene nanosheets can be collected as shown in Figs. S1–S4.

### Preparation of PU@PDA

The dilute HCl was dripped to 200 mL of Tris buffer (1.2 g L^−1^) until the pH ≈ 8.5. DA and PU sponges (1:1) were immersed into the mixture and slowly stirred for 24 h at 60 ℃. The PU covered by PDA (PU@PDA) was cleaned cyclically with ethanol and deionized water 3 times to eliminate self-polymerized DA and obtained by drying in the oven at 70 ℃ for 1 h [11, 17].

### Preparation of MXene/Graphene/PU@PDA (MGP) Composite Sponges

The PU@PDA sponge was dipped into NFC/MXene/Graphene solution with the given concentration and then compressed several times to adsorb completely, followed by vacuum drying at 60 ℃. MXene/Graphene/PU@PDA composite sponges with different concentrations were named as MGPx. When the concentration was higher than 100 mg mL^−1^, a small number of nanosheets would precipitate on the surface after the sponge absorption. Therefore, NFC/MXene/Graphene dispersion (100 mg mL^−1^) was cyclically dipped to further increase the filler loading. MXene/Graphene/PU@PDA composite sponges with different times of dipping were named MGP100-x.

### Preparation of MGP/PEG (MGPP) Composites

The MGPP composites were fabricated using vacuum-assisted impregnation of PEG. The corresponding MGPPx or MGPP100-x were permeated into molten PEG at 75 ℃ overnight in a vacuum oven. The content was acquired by weighing the quality of the PU@PDA, MGP sponges, and MGPP composites after PEG infusion.

### Characterizations

The microstructure of PU, PU@PDA, and MGP composite sponges was observed by scanning electron microscopy (SEM, JSM-6700F, JEOL, Japan). The mechanical properties of MGP composite sponges and MGPP composites were performed by DMA (TA Instruments, Q850). The thermal conductivity (*λ*, W m^−1^ K^−1^) can calculate as follows:

1 $$\lambda \, = \, \rho \, \times C_{p} \times \, \alpha$$

where a NETZSCH LFA447 NanoFlash was applied to measure the thermal diffusivities (*α*, mm^2^ s^−1^) of the composites at room temperature, *C*_*p*_ (J g^−1^ K^−1^) is collected by DSC (TA, Q20) and *ρ* (g cm^−1^) means density. The thermal conductivity enhancement can be calculated as follows:

2 $$TCE\;(\%)=\frac{TC - {TC}_{0}}{{TC}_{0}}$$

where *TC* and *TC*_0_ are the thermal conductivity of the composites and pure polymer matrix or MGPP0 composite, respectively. A TA Q500 HiRes Thermogravimetric analyzer was applied to perform thermogravimetric analysis (TGA) with a heating rate of 10 °C min^−1^. For atomic force microscope (AFM) and scanning thermal microscopy (SThM), Park XE8 with the conductivity contrast mode (CCM) was employed [29, 30]. Thermal mapping signals detected the change in probe current while recording the fluctuation of the surface at the same time. DSC (TA, Q20) tested the thermal properties during phase transition. Enthalpy efficiency (λ) and relative enthalpy efficiency (η) can be calculated as follows:

3 $$\uplambda =\frac{{\Delta H}_{m(\mathrm{MGPP})}}{{\Delta H}_{m(\mathrm{PEG})}}\times 100\%$$

4 $$\eta = \frac{{\Delta H_{m(MGPP)} }}{{\Delta H_{m(PEG)} \times w}} \times 100\%$$

where Δ*H*_m(MGPP)_ and Δ*H*_m(PEG)_ represent the melting enthalpy of MGPP composites and pure PEG, respectively, and *w* represents the mass percentage of PEG in MGPP composites. The heat transport was recorded by an IR thermal imaging spectrometer (Optris PI400, Germany). A PNA-N5244A vector network analyzer from Agilent (Cary, NC) was employed to receive the EMI SE over the X band. From the scattering coefficients *S*_11_ and *S*_21_, reflection parameter (*R*), transmission parameter (*T*), absorption parameter (*A*), total EMI SE (*SE*_T_), reflection loss (*SE*_R_), absorption loss (*SE*_A_), and multiple internal reflection loss (*SE*_M_) of electromagnetic waves can be obtained by the following formula [20, 24, 31]:

5 $$T = \left| {S_{21} } \right|^{2} \,{\text{and}}\,R = \left| {S_{11} } \right|^{2}$$

6 $$R+T+A=1$$

7 $${SE}_{T}=-10\,\mathrm{log}T$$

8 $${SE}_{R}=-10\,\mathrm{log}\left(1-R\right)$$

9 $${SE}_{A}=-10\,\mathrm{log}\left[T/(1-R)\right]$$

10 $${SE}_{T}={SE}_{R}+{SE}_{A}+{SE}_{M}$$

When *SE*_T_ is greater than 15 dB, *SE*_M_ could be ignored. High-resolution transmission electron microscopy (HRTEM, JEM-2010F, JEOL, Japan), Fourier transforms infrared (FTIR, AVATAR370, Nicolet, USA) and X-ray diffraction (XRD, D/MAX-2200/PC, Rigaku, Japan) were applied.

## Results and Discussion

### Construction of the MGP Conductive Networks

**Fig. 1:**
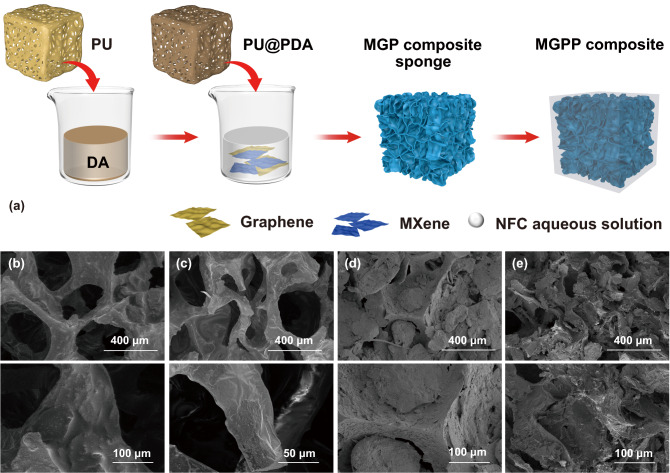
**a** Process for fabricating MGPP composites. Surface SEM images of **b** PU, **c** PU@PDA, **d** MGP100 composite sponges, and **e** MGP100-3 composite sponges and their corresponding magnification images

Figure 1a shows a schematic of the MGPP composite prepared by water-based dip coating and surface treatment. It is difficult to uniformly coat the aqueous solution of MXene and graphene due to the hydrophobicity of the PU sponge. Using the self-polymerization of dopamine to form a polydopamine-modified PU sponge, the PU@PDA sponge with good hydrophilicity can be obtained (Fig. S5). The PU@PDA emerges the blue shift of the hydroxyl (–OH) stretching band, which wave number is from 3390 to 3371 cm^−1^ (blue shift 19 cm^−1^) compared with the PU sponge. The blue shift confirms the formation of hydrogen bond interaction. Moreover, the water contact angle is from 120.6° to 97.8°, which affirms the improved hydrophilicity from PU to PU@PDA sponge. The PU@PDA was immersed in NFC/MXene/Graphene dispersions of different concentrations and pressed repeatedly until the excess liquid could not be retained in the sponge. An appropriate concentration of binder was introduced to increase the hydrogen bonding interaction to ensure that the MXene and graphene nanosheets would not easily fall off from the network (Fig. S6). The introduction of NFC can effectively increase the viscosity of the dispersion. Therefore, the dispersion can be absorbed into the micropores of the PU sponge without any leakage. NFC, as an environmentally friendly green dispersant, has a large number of hydroxyl groups, which can increase the hydrogen bond interaction at the same time [32, 33]. Finally, PEG was infused into the MGP composite sponge to obtain the MGPP composite. When the concentration of the dispersion reaches 100 mg mL^−1^, a small amount of MXene and graphene nanosheets will accumulate on the surface of the PU sponge during the coating. Therefore, cyclic dipping was applied to increase the filler content in the MGPP composite. As the number of cycles increases, growing solid residues remain on the surface. Figure 1b, c demonstrates that PU and PU@PDA sponges have similar porous structures, but the modified PU@PDA makes the original smooth PU surface rough. After dip-coating the dispersion, MXene and graphene nanosheets can be uniformly deposited on the PU network, and the sheet-like structure can be observed in Fig. 1d. As the number of cycles of dipping increases, the open micropores of the PU@PDA sponge are filled with MXene and graphene nanosheets. Figure 1e indicates that MXene and graphene will overlap and cover the original open micropores when the content of MXene and graphene nanosheets further increases. The cross-sectional SEM images of the MGP composite sponge can draw the same conclusion (Fig. S7). MXene and graphene nanosheets can fully cover the PU@PDA sponge, decreasing the pore size in the sponge and making a denser structure. After comparing melamine foam and PU sponge, the choice of PU sponge as the soft template for constructing a 3D interconnected network is precisely caused by this feature of the MGP composite sponge (Fig. S8). The open microporous structure of the PU sponge can provide MXene and graphene nanosheets with more space to overlap on the original skeleton to form a denser MGP composite sponge. The capillary effect brought by this dense structure is beneficial to avoid voids in the polymer composites during the subsequent vacuum infusion process.

### Mechanical Property of the MGP Composite Sponges

**Fig. 2:**
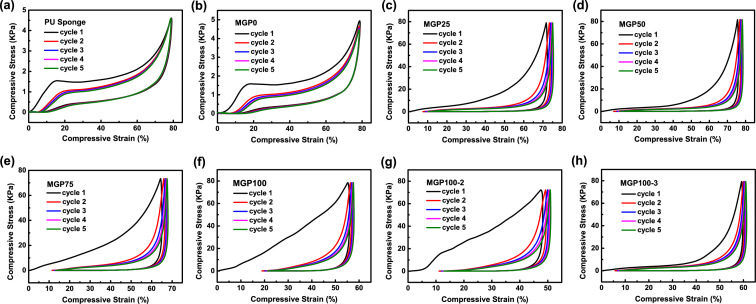
The compressive curves of **a** PU and **b** MGP0 sponge at the strain of 80% for 5 cycles. The compressive curves of **c** MGP25, **d** MGP50, **e** MGP75, **f** MGP100, **g** MGP100-2, and **h** MGP100-3 composite sponges at the same stress of 16 N for 5 cycles

The 3D PU sponge endows the MGP composite sponges with excellent mechanical properties [34], especially when the PU and PU@PDA sponges can recover their original shape under 80% strain compression. As shown in Fig. 2a, the PU sponge can recover to its original shape after five cycles of compression. Compared with Fig. 2b, the PU@PDA (MGP0) sponge maintains the compressibility of the PU sponge, while the stress of PU@PDA is slightly improved. This is mainly attributed to the interfacial effect generated by the PDA modification on the PU surface. Figure 2c–h shows the compressive stress curves of MGP composite sponges loaded with different contents of MXene and graphene mixed fillers. The compressibility of the MGP composite sponge is limited due to the MXene and graphene nanosheets being tightly coated on the surface of the PU@PDA, while the stress of the composite sponge is increased. Under the same 16 N stress, the MGP composite sponges exhibited reversible compression performance under multiple cycles. However, the MXene and graphene nanosheets attached to the surface of the PU@PDA hindered the composite sponge from quickly recovering to its original state after compression. Under the same stress, the strains of MGP composite sponges are different. MGP50 shows a maximum strain of nearly 80%, while MGP100-2 shows a minimum of 50%. This shows that the NFC/MXene/graphene dispersion of 100 mg mL^−1^ has been able to completely coat the surface of the PU@PDA sponge, limiting its rapid recovery after deformation. Although the MGP100-2 sponge can still partially recover after deformation, it cannot fully recover before compression. Surprisingly, the recovered strain for MGP100-3 is nearly 60%, while the increasing concentration of the NFC/MXene/graphene dispersion resulted in the decreasing strain. During the third cycle of dip coating, the growing content of NFC is immersed into the composite sponge due to the reduced nanosheets adsorption of the composite sponge, which may cause the growing content of NFC in the composite sponge. TGA analysis exhibits weight loss between 300 and 400 °C, which belongs to the decomposition of NFC (Fig. S10). NFC has excellent mechanical properties and can enhance the MGP for excellent compressive recovery [35]. At the same time, the increase in NFC improves the hydrogen bond interaction, and the existence of hydrogen bonds further leads to increased strain [17].

### Thermal Management Properties of the Polymer Composites

**Fig. 3:**
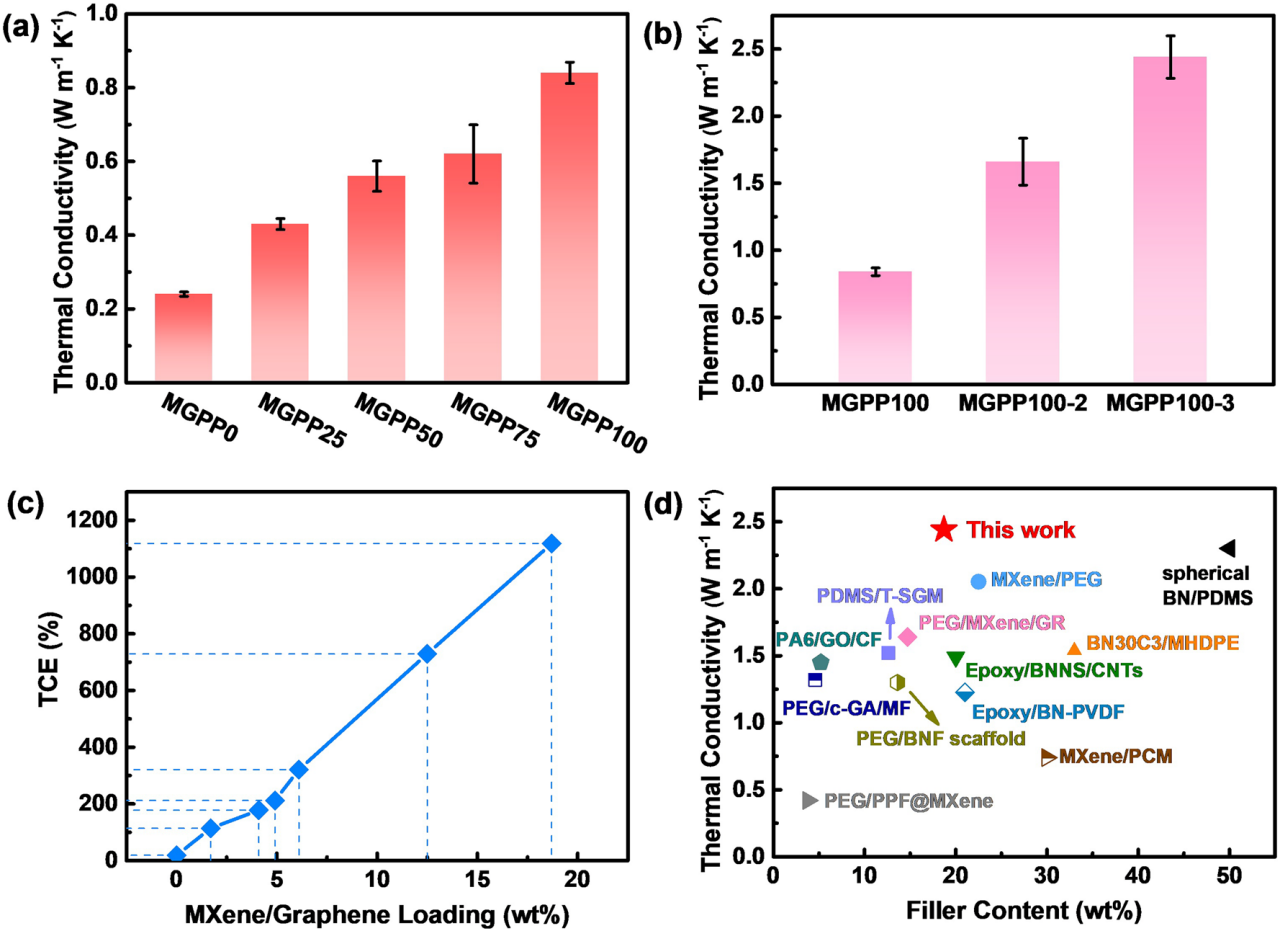
Through-plane thermal conductivity of MGPP composites with different **a** concentration and **b** number of cycles. **c** TCE with MXene/graphene loading. **d** Comparison of thermal conductivity of different 3D network filled composites: spherical BN/PDMS [54], MXene/PEG [55], PEG/MXene/GR [36], PA6/GO/CF [37], BN30C3/MHDPE [56], Epoxy/BNNS/CNTs [18], PDMS/T-SGM [57], PEG/c-GA/MF [58], Epoxy/BN-PVDF [59], PEG/BNF scaffold [29], MXene/PCM [60], CCA@rGO/PDMS [51], PEG/PPF@MXene [61]

Figure 3a shows through-plane thermal conductivity with increasing concentration of NFC/MXene/graphene mixed dispersion. Figure 3b shows the through-plane thermal conductivity with increasing coating times. The thermal diffusivity of the MGPP sample can obtain in Fig. S9a. The correspondence between thermal conductivity enhancement (TCE) and filler content in MGPP composites can be obtained in Fig. 3c. When the content of MXene and graphene amounts to 18.7 wt%, the through-plane thermal conductivity of MGPP100-3 composite reaches the maximum value of 2.44 W m^−1^ K^−1^ and reaches 1118% compared to that of pure PEG (~ 0.20 W m^−1^ K^−1^) and 920% compared to that of MGPP0 (Fig. S9b). Figure 3d shows the thermal conductivity comparison of this work and other 3D network-filled polymer matrix composites. PU sponge as a 3D structural soft template, with a facile solution coating method, is an effective strategy to achieve thermal conductivity improvement at relatively low filler addition. TGA analysis is also commonly applied to characterize the thermal stability of MGPP composites (Fig. S10). The decomposition starts at about 340 °C and completes at about 440 °C. A slight weight loss can be observed in MGPP100-2 and MGPP100-3 before 340 °C in the inserted image, which could be attributed to the increased NFC content. Figure S10b shows that the residual amount of MGPP0 is approximately 0 after completely decomposing, and the residual amount of MGPP composites increases with the growing content of MXene and graphene content. The increased NFC content of MGPP composites improves the interfacial compatibility of MGP composite sponges and PEG, which can be confirmed by the blue shift of the hydroxyl (–OH) stretching band of MGPP100, which wave number is from 3474 to 3422 cm^−1^ (blue shift 52 cm^−1^) compared with pure PEG (Fig. S4).

**Fig. 4:**
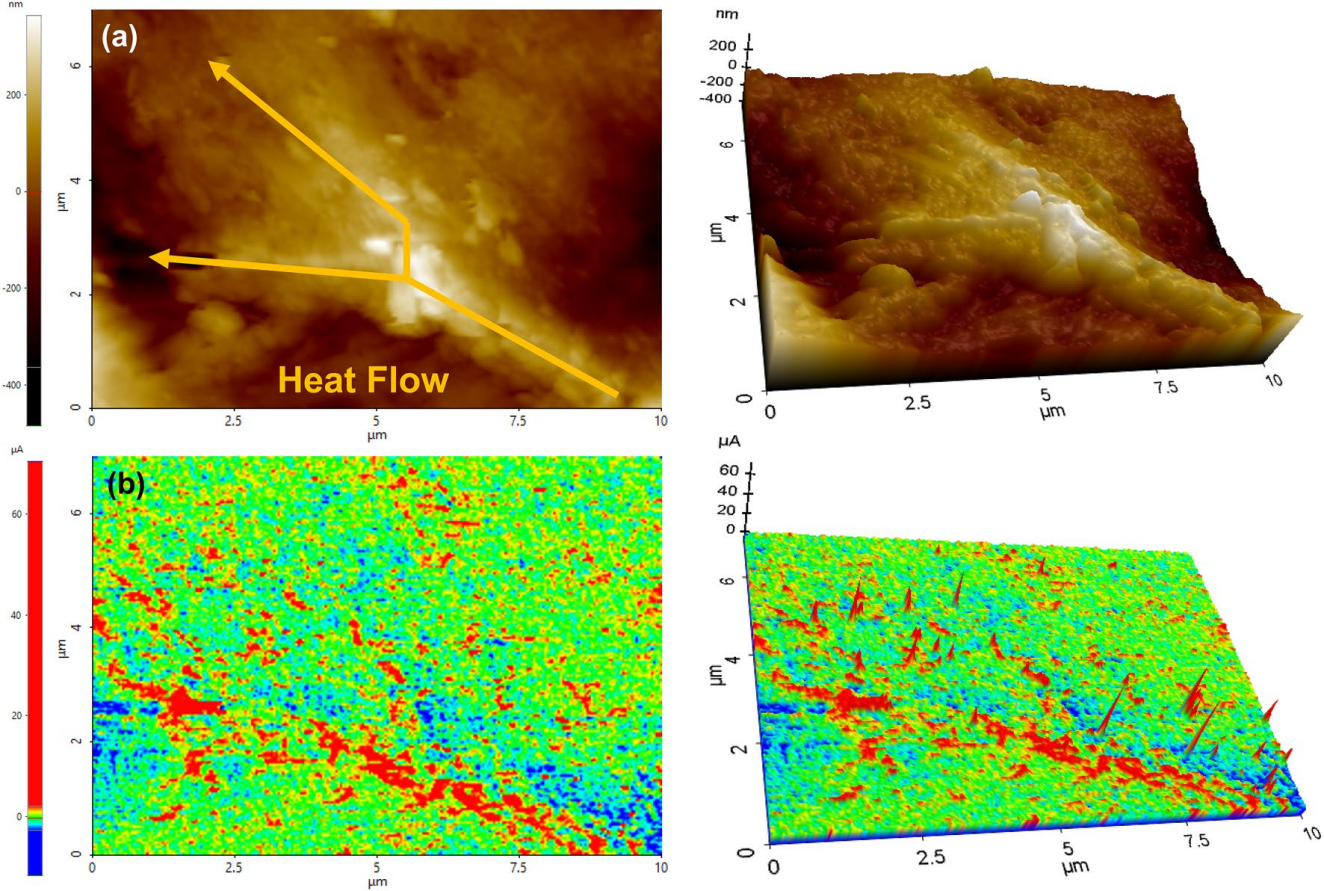
**a** Topography and **b** SThM and their relevant 3D graphics of MGPP100-3 composite. The scanning area is 10×7 μm^2^

Figure 4 presents the topography and scanning thermal microscope (SThM) graphics and their relevant 3D maps of MGPP100-3 composite. The white bright areas in the topography map correspond to the protrusions on the surface, and the black dark areas correspond to the depressions of the surface. As shown in Fig. 4a, the distinct dendritic protrusion can be observed, corresponding to the 3D interconnected network of the MGP composite sponge. The agglomeration of the PEG molecular chain results in a 200 nm bulge. The signal intensity of MGPP0 is approximately 0 μA after normalization in Fig. S11. MGPP0 does not introduce high thermal conductivity functional fillers MXene and graphene, indicating the current signal intensity generated by the polymer, which corresponds to the green area. In Fig. 4b, the green signal area corresponds to the part of the PEG matrix in the MGPP100-3 composite. Due to the limited thermal conductivity of the PEG, the charge cannot be quickly converted into heat dissipation. Therefore, the probe would not be required a continuous input charge. Accordingly, the red color corresponds to the electrically and thermally conductive 3D interconnected network pathway that produces a strong current signal. The current input by the probe is rapidly converted into heat energy to dissipate along the MGPP composite network pathway. Therefore, more current needs to be continuously input to achieve charge balance. The existence of the blue negative current signal area is caused by the fluctuation of the current signal with the roughness of the composite surface topography. After normalization, the relatively low-intensity current signal becomes negative. This apparent current difference enables us to visually observe the heat transport behavior on the surface of the MGPP composite, where the largest current signal difference reached nearly 60 μA [36, 37].

PEG will melt at above 70 °C greatly limiting the application of PEG-based composites in high-temperature environments. As shown in Fig. S12a, MGPP composites were manufactured into thermally conductive blocks of the same size, placed on a continuously heating plate and heated to 90 ℃. Infrared thermal imaging in Fig. S12b demonstrated that the filling of the 3D MGP composite sponge could ensure the MGPP composite will not leak at a temperature above melting point of pure PEG. Figure S12c records the variation of surface temperature. The surface temperature of the MGPP100-3 composites was 1–2 °C higher than that of the MGPP100 and MGPP100-2 under the same heating time. The MGPP100-3 composite shows the most uniform heat transfer performance in the process of continuous heating.

**Fig. 5:**
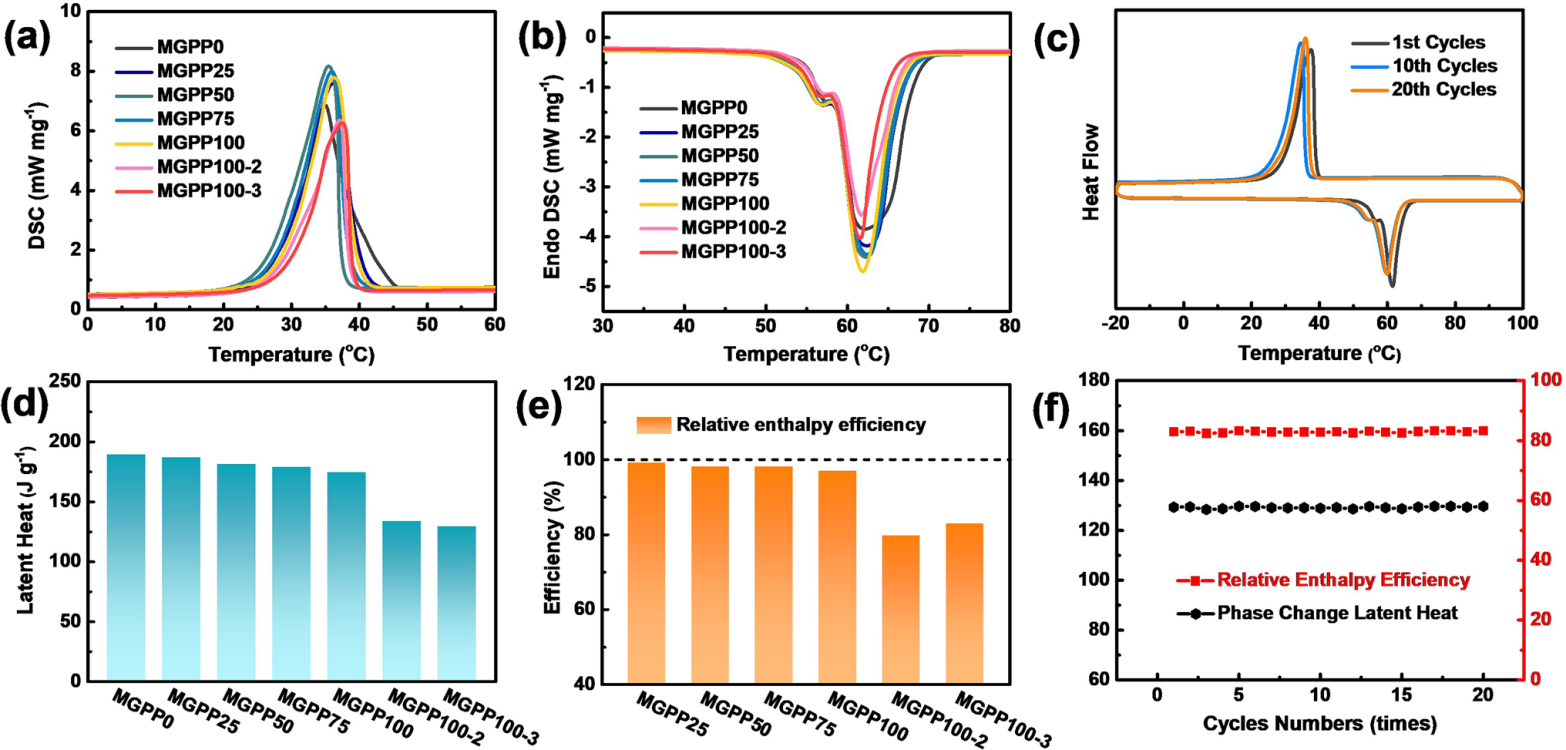
**a** Cooling process and **b** heating process of DSC curves of MGPP composites. **c** DSC curves of MGPP100-3 with different numbers of thermal cycles. **d** Phase change latent heat and **e** relative enthalpy efficiency of MGPP composites. **f** Phase change latent heat and relative enthalpy efficiency of MGPP100-3 with different numbers of thermal cycles

DSC can heat the sample to a temperature beyond its phase change to measure the heat change before and after the phase change, which is an important method in the evaluation of the phase change latent heat [38]. Figure 5a, b shows the exothermic and endothermic processes of the DSC curves of MGPP composites, respectively. Figure S13a shows the DSC curve of PEG, and Tables S1-S2 show data obtained from the DSC measurement. The initial melting and crystallization temperatures of PEG were 56.66 and 37.38 °C, and the final temperatures were 65.46 and 24.57 °C, the ranges of melting and crystallization temperatures were 8.8 and 12.81 °C, respectively. The initial melting and crystallization temperatures of the MGPP0 were 58.68 and 40.44 °C, the final temperatures were 68.11 and 24.41 °C, and the ranges were 9.43 and 16.03 °C, respectively. Compared with pure PEG, the melting and crystallization temperature range of MGPP0 are broadened. The process of the polymer chain from melting to crystallization is divided into crystal nucleation and crystal growth. The addition of PU provides better crystal nucleation conditions, so the crystallization temperature difference becomes wider, leading to the melting temperature difference becoming wider too. MXene and graphene can accelerate the heat exchange, thereby affecting the melting temperature [39]. The range of melting and crystallization temperatures become narrow. The growing filler content can help more heat to be stored as latent heat in the MGPP composites, thereby reducing the apparent melting temperature. Figure 5c displays DSC curves of MGPP100-3 with 1st, 10th and 20th thermal cycles number, which appears to be a slight decrease in *T*_m_ and *T*_c_ after multiple cycles. Figure 5d illustrates that the phase change latent heat decreases with the increasing addition of MXene and graphene. MGPP0 exhibits the maximum latent heat of 189.43 J g^−1^, while MGPP100-3 exhibits the latent heat of 129.32 J g^−1^. When the loading of MXene and graphene is increased to 18.7 wt%, the phase change latent heat is reduced by approximately 32% and the thermal conductivity is raised to 2.44 W m^−1^ K^−1^, thus providing a compensating effect. To evaluate the thermal energy storage capacities of MGPP composites, their enthalpy efficiencies (*λ*) and relative enthalpy efficiencies (*η*) were calculated. Generally, the superior the *λ* and *η* values, the greater the thermal energy storage capacity. Figure 5e gives the *η* value of the MGPP composites, and Fig. S13b gives the *λ* value of the MGPP composites. With the increase in MXene and graphene content, the η values of MGPP composites are 99.3%, 98.8%, 98.3%, 97.1%, 79.8%, and 83.0%, respectively, showing superior thermal energy storage ability. Figure 5f gives the corresponding phase change latent heat and η of MGPP100-3 with different numbers of thermal cycles. After multiple cycles, neither phase change latent heat nor relative enthalpy efficiency was observed to change significantly. The η values of MGPP100-3 remain at about 83%. This certificates the outstanding thermal reliability and reusability of the MGPP composites.

### Thermodynamic Behavior of the MGPP Composites

**Fig. 6:**
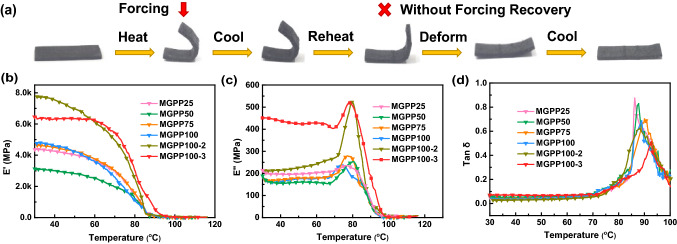
**a** The digital images display the heat-sensitive shape memory feature of MGPP composite. **b** The storage modulus, **c** loss modulus, and **d** loss factor tan δ curves with temperature

The thermomechanical property of the thermally sensitive shape memory material MGPP100-3 composite is given in Fig. 6a. In the beginning, heating and softening the MGPP100-3 spline, and then maintaining the temperature and deforming under a given external force. The next step, cooling down under the loading of external force. When the spline is solidified, the external force is removed and the spline maintains its temporary shape. Finally, the MGPP100-3 spline can spontaneously recover its shape when it is reheated. Due to the reduction in the heated area after deformation, the spontaneous recovery process of the spline will spend a longer time. After reheating, the spline initially has a rapid shape recovery (Movie S1), and then the speed of recovery will gradually slow down (Movie S2). After a while, the shape of the MGPP100-3 spline does not change, which spline is slightly cocked at this time due to the melting of PEG. After cooling and solidifying, the MGPP100-3 composite returned to a shape similar to its original condition. The above series of processes is a cycle in the shape memory process, indicating that the MGPP composites have reversible thermomechanical properties [25]. This dynamic thermomechanical property was shown to be utilizable in the construction of thermal diodes [40].

DMA can characterize the properties of materials by measuring the state of molecular motion, which determines the stiffness and damping. The storage modulus (*E*’) represents the mechanical energy stored internally by the material and thereby relates to the stiffness and shape recovery of the polymer. The loss modulus (*E*’’) represents the damping behavior, which is the ability of the polymer to disperse mechanical energy through internal molecular motion. When a variable amplitude sinusoidal alternating stress is applied to the sample, the strain of the viscoelastic sample will lag a certain phase angle *δ* correspondingly, and the resulting phase difference tan δ is called the loss factor. Tan *δ* is the ratio of *E*’’ and *E*’. Figure 6b–d presents the curves of *E*’, *E*’’ and tan *δ* for MGPP composites. The relatively flat region of Fig. 6b at lower temperature corresponds to the glassy state of the composite. The glass transition starts from a downward curve, and the sharp drop is caused by the significant softening of the PEG. After reaching the melting point, MGPP100-3 showed the largest *E*’, about 6240 MPa. The maximum value of *E*’’ corresponds to the beginning of the segmental motion of the polymer chain. When the damping phenomenon occurs, the mechanical energy is converted into heat dissipation through the internal friction of the molecular chain. Figure 6c shows that the *E*’’ of MGPP100-3 and MGPP100-2 reach the maximum value of 519 and 523 MPa around 80 °C, respectively. Figures 6d and S14 show the variation and maximum value of the tan *δ* of MGPP composites with temperature, respectively. The tan δ decreased from 0.87 to 0.52 as the filler content of the composites increased. Due to the growing content of MXene and graphene increasing the cross-linking point inside the composite, the cross-linking density is raised and the mobility of molecular chains has deteriorated. The internal friction caused by molecular chain slippage lessens, which is manifested as the reduction of tanδ. The change of the tan δ also can track the viscoelasticity of composites. The tanδ rises initially and then drops rapidly, indicating that the composites have changed from elastic state to viscoelastic and eventually to soft elasticity.

### EMI Shielding Performance of the MGPP Composites

**Fig. 7:**
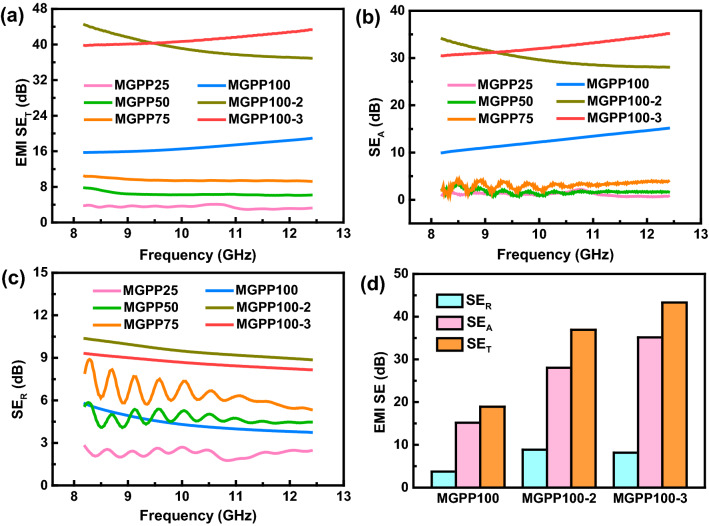
**a** *SE*_T_, **b** *SE*_A_, and **c** *SE*_R_ of MGPP composites at X bandwidth. **d** EMI shielding performance comparison of MGPP composites at 12.4 GHz

MGPP composites not only have enhanced thermal management performance but also have excellent EMI shielding performance. Figure 7a–c corresponds to the *SE*_T_, *SE*_A_, and *SE*_R_ values of the MGPP composite at the thickness of 2.4 mm, respectively. With the increasing content, MGPP composites exhibited gradually improved electromagnetic shielding performance. The shielding effectiveness of MGPP100-3 reaches a maximum value of 43.3 dB at the *X*-band, which has reached the general standard for commercial applications (~ 20 dB). The total shielding effectiveness (*SE*_T_) consists of the absorption (*SE*_A_) and reflection (*SE*_R_) of the composite. According to Fig. 7b, c, the devotion of SE_R_ of MGPP25, MGPP50, and MGPP75 is more considerable than *SE*_A_. The reason for this phenomenon is mainly because coating a low-concentration NFC/MXene/graphene dispersion is difficult to completely cover the PU sponge, providing an approach for electromagnetic wave leakage. However, the devotion of MGPP100, MGPP100-2, and MGPP100-3 is mainly absorption shielding effectiveness. Due to the reflection of electromagnetic waves leading to secondary pollution, shielding materials based on absorption are the most ideal proposal. Figure 7d shows the electromagnetic shielding performance at 12.4 GHz. The percentages of *SE*_A_ occupied *SE*_T_ of MGPP100, MGPP100-2 and MGPP100-3 were 80.4%, 75.9% and 81.3%, respectively. The MGPP100-3 composite can keep more than 80% of the incident electromagnetic wave inside the structure and convert it into heat energy dissipation. Owing to the excellent thermal conductivity of MGPP100-3, the heat converted from electromagnetic waves can be dissipated in time, making it an ideal multifunctional material for the preparation of smart electronic devices.

Table 1 compares the MGPP100-3 composite with other polymeric composites reported in the literature [20, 36, 41–52]. Conductive metallic materials were initially widely used for electromagnetic protection, but their poor corrosion resistance and inflexibility limit their applications. The reinforced metallic fillers such as silver nanowires or particles are applied in practical research. Carbon-based fillers represented by graphene and MXene have comparable electrical properties to metallic fillers. Therefore, polymer composites containing carbon-based fillers show excellent EMI shielding performance while overcoming many of the defects of metallic fillers. The absolute shielding effectiveness is introduced to exclude thickness (*t*) and density (*ρ*) to characterize shielding effectiveness. SSE/t is more valuable for the contrast of lightweight shielding materials like films and sponges. Simultaneously, SSE excluding the density and *SE/t* excluding the thickness are defined as a reference for comparing the shielding effectiveness of composites. Since the density of composites is relatively close, *SE/t* is a worthy reference to evaluate shielding effectiveness. The MGPP100-3 composite still shows obvious superior performance under the condition of excluding the thickness factor at the same X band.

**Table 1:** Comparison of EMI shielding of polymer matrix composites

Materials	Thickness (mm)	EMI SE (dB)	Frequency (GHz)	Density (g cm^−3^)	Filler Content	SE/t (dB cm^−1^)	SSE/t (dB cm^2^ g^−1^)	Refs.
Epoxy/wood-Derived carbon	2	27.8	8.2–12.4	1.17	7 vol%	139	119	[41]
PEG/MXene/GR	2.5	36	8.2–12.4	1.27	14.7 wt%	144	113	[36]
Epoxy/Fe_3_O_4_/TAGA	3	35	8.2–12.4	–	2.7 wt%	117	–	[47]
PDMS/Graphene	5	42	8.2–12.4	–	0.42 wt%	84	–	[43]
rGO@FeNi/EP	–	46	8.2–12.4	–	2.1 wt%	–	–	[52]
SiCnw@MXene PVDF-HFP	1.8	26.85	8.2–12.4	–	30 wt%	149	–	[50]
Fe_3_O_4_/PI	0.085	34	8.2–12.4	–	23.8 wt%	400	–	[49]
CCA@rGO/PDMS	/	51	8.2–12.4	–	3.05 wt%	–	–	[51]
SCF/EVA	3.5	34	8–12	–	30 phr	97	–	[46]
PMMA/GNPs/MWCNTs	2.5	36	8–12	0.6	/	144	240	[44]
CMP/CS	8	29.3	8.2–12.4	0.15	/	37	244	[48]
CB/UHMWPE	2.1	33.3	8.2–12.4	–	15 wt%	159	–	[45]
rGO/PS	2.5	45.1	8.2–12.4	–	3.47 vol%	180	–	[42]
Ni@MF/CNT/PBAT	1.8	29.5	8.2–12.4	–	–	164	–	[20]
MGPP100-3	**2.4**	**43.3**	**8.2–12.4**	**1.36**	**18.7 wt%**	**180**	**133**	**This Work**

### Mechanical Property of the MGPP Composites

**Fig. 8:**
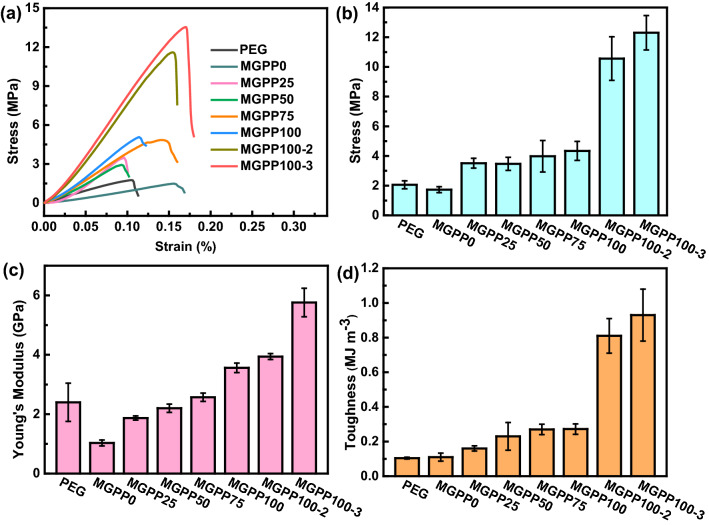
**a** Strain–stress curve, **b** stress, **c** Young’s modulus, and **d** toughness of MGPP composites

The mechanical properties of composites are also an important factor affecting their practicality [53]. Figure 8 explores the mechanical properties of MGPP composites and PEG. The enlarged strain–stress curve at the range of 0–0.05% strain can be obtained in Fig. S15. Compared with PEG, the filling of PU@PDA (MGPP0) reduces the original stress and Young's modulus of PEG, while improving the toughness of PEG. The poor interfacial compatibility between PU@PDA and PEG is prone to internal structural defects leading to fracture. The introduction of MXene and graphene resulted in improved stress, Young's modulus, and toughness of MGPP composites, which increased with the growing addition of MXene and graphene content. The enhanced mechanical properties can be attributed to (1) PDA acts as a spring-like binder to fix MXene and graphene nanosheets on the PU surface to provide more interlocking sites to resist deformation, (2) the increasing of MXene and graphene content strengthens the interfacial compatibility between PU@PDA and PEG, causing the better mechanical properties of MGPP composites, (3) NFC inherently has a large number of hydroxyl groups and excellent mechanical properties. Therefore, the introduction of NFC reinforces the hydrogen bond interaction, which can effectively improve endurance. MGPP100-3 composite exhibits the most superior mechanical properties, with a stress of 12.3 MPa, Young's modulus of 5.76 GPa, and toughness of 0.93 MJ m^−3^. Moreover, the stress, Young's modulus, and toughness of the MGPP100-3 composites have been upgraded 6 times, 4 times, and 9 times compared with pure PEG, respectively. The mechanical properties of PEG are improved through the filling of MGP composite sponges, thus expanding their potential applications as multifunctional composites for thermal conductivity and electromagnetic shielding.

## Conclusion

In this work, the utilization of the soft template with the modification of polydopamine and a dispersion dip-coating approach effectively constructed the 3D thermally and electrically conductive interconnected network structures of the multifunctional polymer composites. When the loading of MXene and graphene nanosheets reaches 18.7 wt%, the improved through-plane thermal conductivity of polymer composite is 2.44 W m^−1^ K^−1^, which is 1118% superior than that of the pure polymer matrix. The electromagnetic shielding effectiveness of the sample reaches 43.3 dB, which has compassed the general standard for commercial applications. Furthermore, the Young’s modulus of the sample have advanced 4 times. This eminent performance evidences polymer composites can be potentially served as innovatory multifunctional materials for applications in smart telecommunications and portable electronic devices.

## Supporting Information

TEM and AFM of MXene and graphene nanosheets; XRD of MAX, MXene, and graphene; FTIR spectra of Ti_3_AlC_2_ MAX, Ti_3_C_2_T_*x*_ MXene, graphene, PEG, NFC, MG/NFC, and MGPP100; FTIR of PU, PDA, PU@PDA, and Water contact angle of PU, PU@PDA; SEM images with PVA and NFC; cross-sectional SEM images of MGPP composites; SEM images with PU and MF foam; thermal diffusivity; DSC curve and DSC data; TGA analysis; topography and SThM of MGPP0; infrared thermal images; Mechanical properties of PEG.

## Supplementary Information

Below is the link to the electronic supplementary material.

Supplementary file1 (MP4 1323 kb)

Supplementary file2 (MP4 610 kb)

Supplementary file3 (PDF 1133 kb)
