# Supplementary material for: Interconnected MXene/Graphene Network Constructed by Soft Template for Multi-Performance Improvement of Polymer Composites
Source: Nanomicro Lett. 2022 Jun 14;14:133. doi: 10.1007/s40820-022-00877-7 (PMC9198158; doi:10.1007/s40820-022-00877-7)
Supplement: Supplementary file 3 — Supplementary file3 (PDF 1133 kb) [file 40820_2022_877_MOESM3_ESM.pdf]

Supporting Information for

Interconnected MXene/Graphene Network Constructed by Soft Template for Multi-Performance Improvement of Polymer Composites

Liyuan Jin[1], Wenjing Cao[1], Pei Wang[1], Na Song[1], and Peng Ding[1, *]

[1]Research Center of Nanoscience and Nanotechnology, College of Sciences, Shanghai University, 99 Shangda Road, Shanghai, 200444, P. R. China

*Corresponding author. E-mail: dingpeng@shu.edu.cn (Peng Ding)

Supplementary Figures and Tables

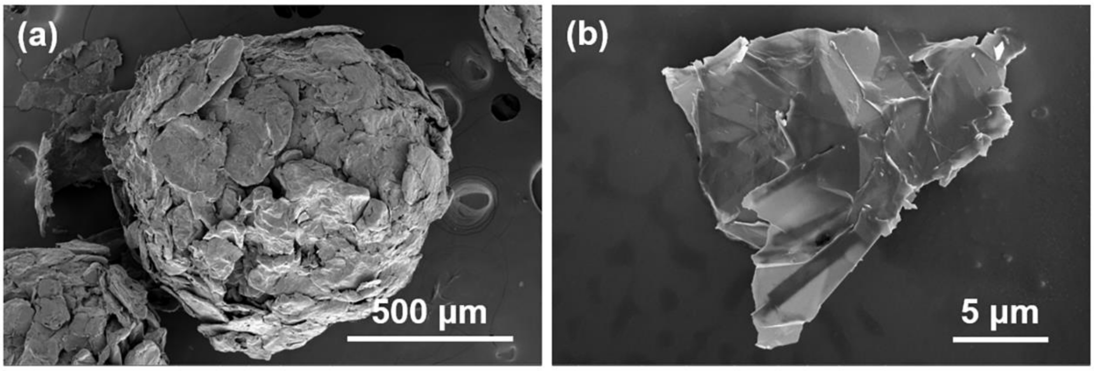

Fig. S1 SEM images of (**a**) spherical-like graphene powder without ultrasonic dispersion and (**b**) Graphene sheets obtained after ultrasonic dispersion

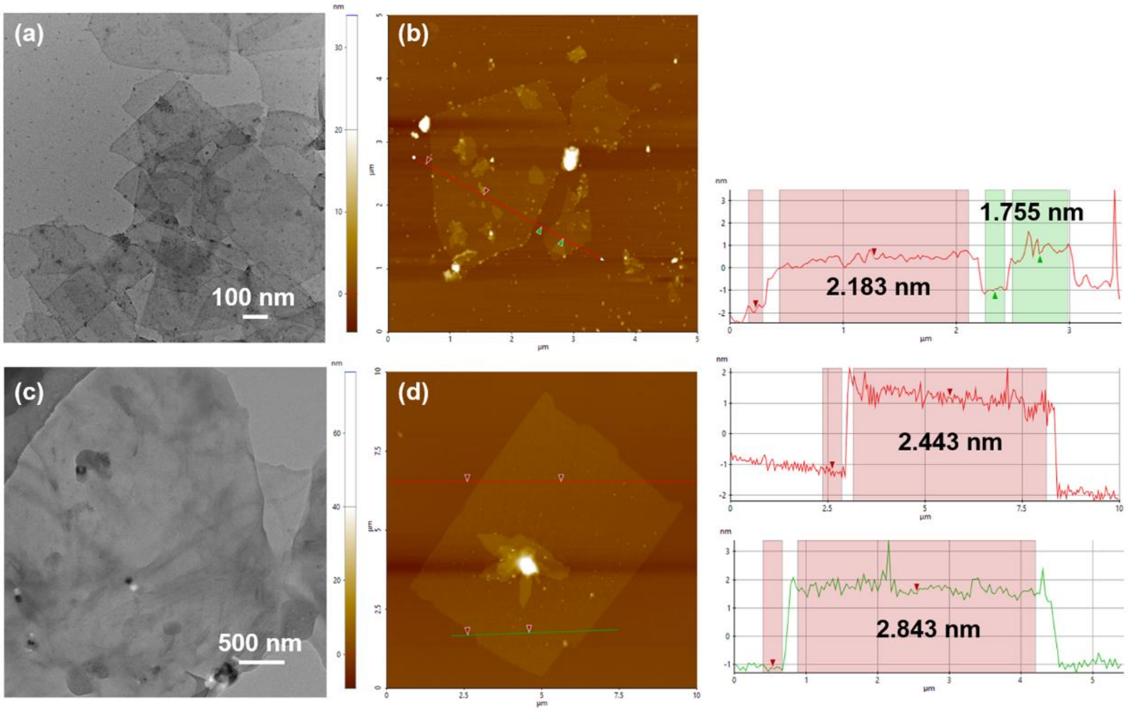

Fig. S2 (**a**) TEM and (**b**) AFM images of MXene nanosheets. (**c**) TEM and (**d**) AFM images of graphene nanosheets

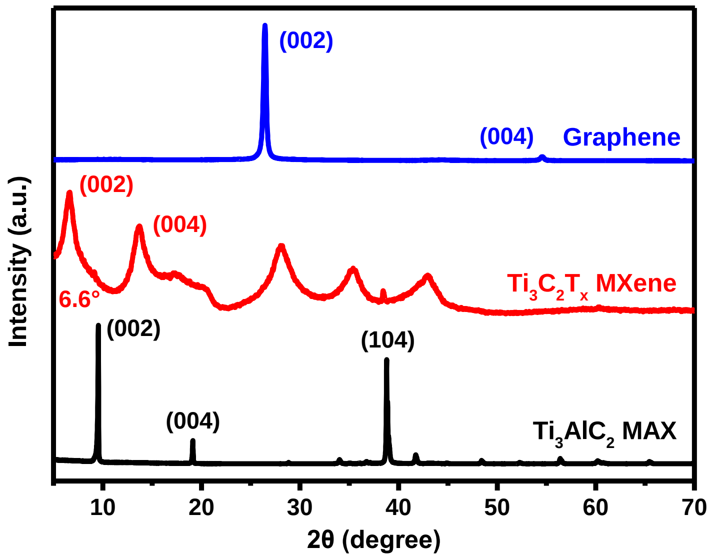

Fig. S3 XRD patterns of Ti_3AlC_2 MAX, $Ti_3C_2T_x$ MXene and graphene nanosheets

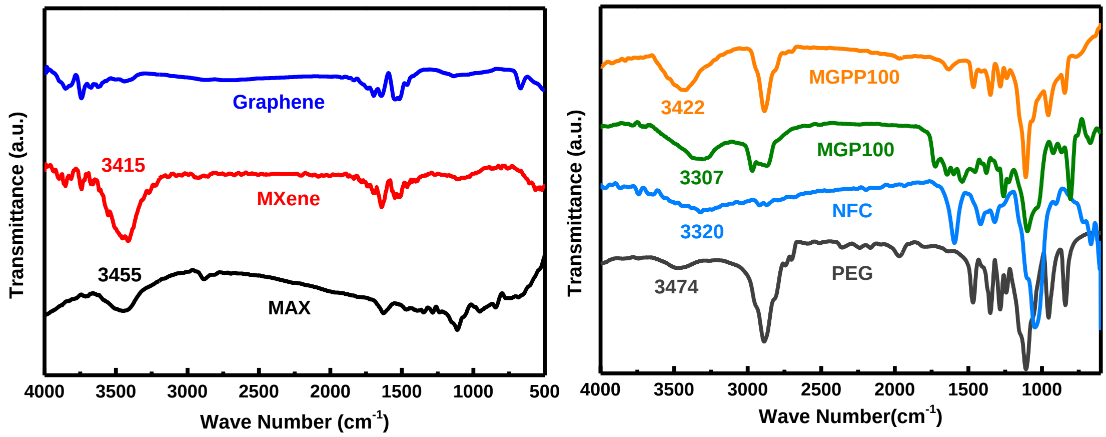

Fig. S4 FTIR spectra of Ti_3AlC_2 MAX, $Ti_3C_2T_x$ MXene, graphene, PEG, NFC, MG/NFC and MGPP100

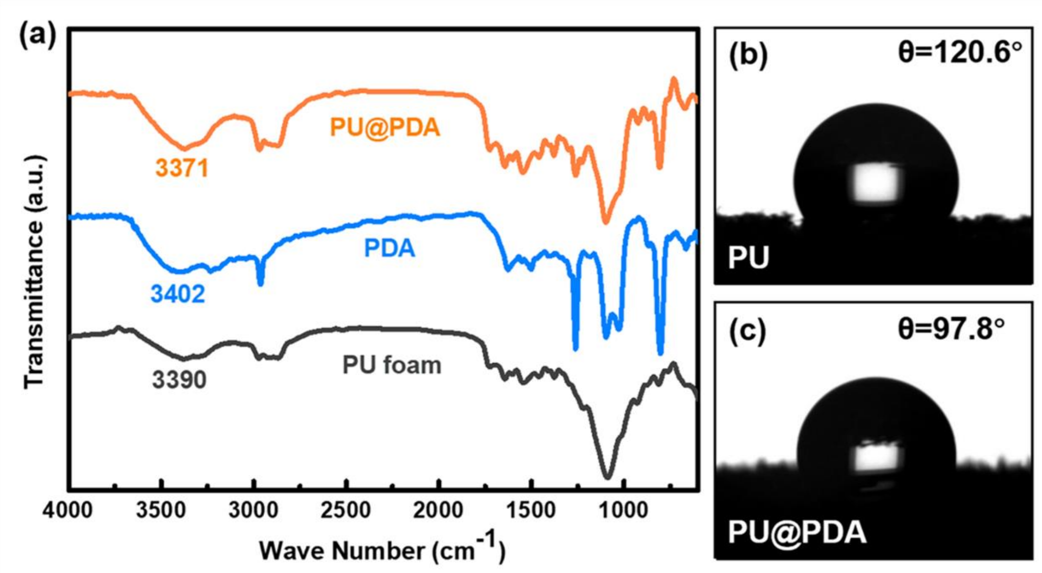

Fig. S5 (**a**) FTIR spectra of PU foam, PDA, and PU@PDA. Water contact angle (WCA) images of (**b**) PU and (**c**) PU@PDA

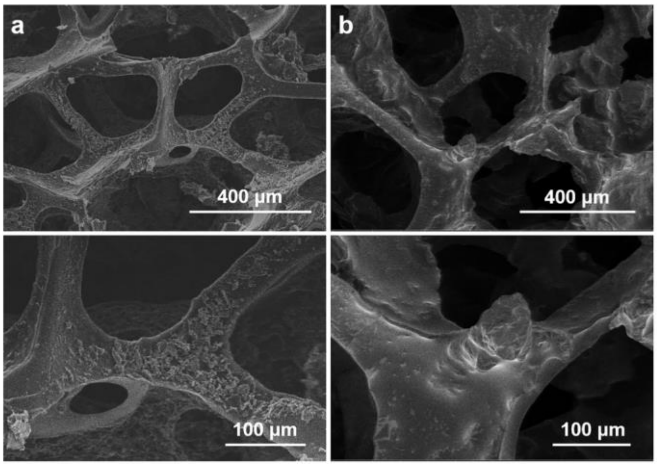

Fig. S6 SEM images of MGP composite foams with (**a**) PVA solution and (**b**) NFC solution and their corresponding magnification images

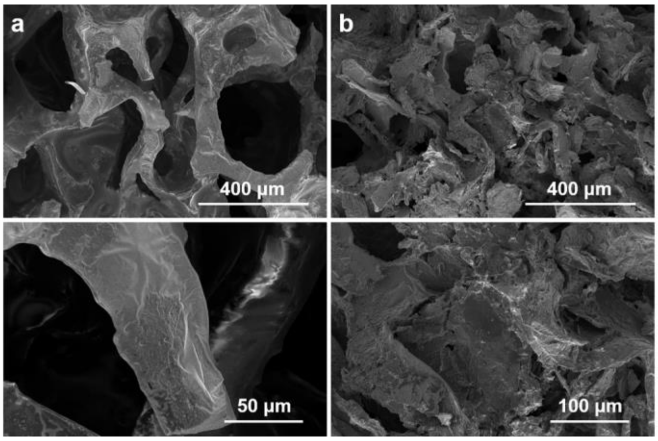

Fig. S7 Cross-section SEM images of (**a**) MGP100 composite foams and (**b**) MGP100-3 composite foams and their corresponding magnification images

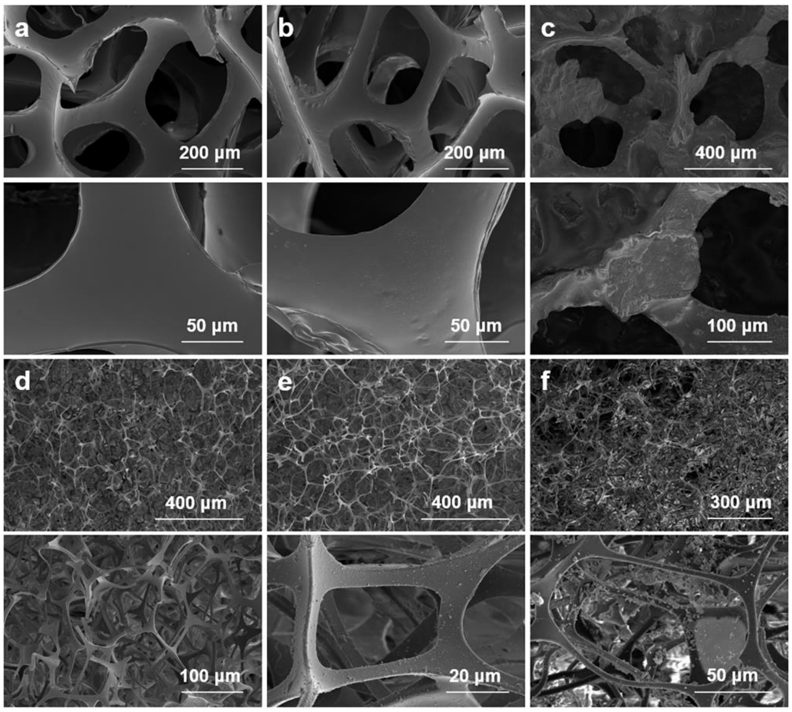

Fig. S8 SEM images of (**a**) PU, (**b**) PU@PDA, (**c**) MGP100 composite foams and (**d**) melamine foam (MF), (**e**) MF@PDA, (**f**) MG/ MF@PDA composite foams

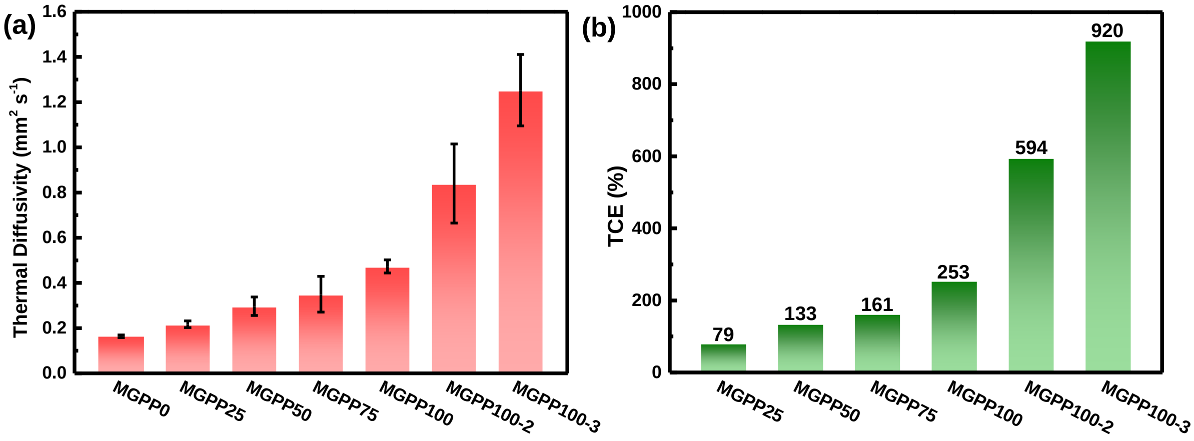

Fig. S9 (**a**) Through-plane thermal diffusivity of MGPP composites. (**b**) TCE of MGPP composites compared with MGPP0

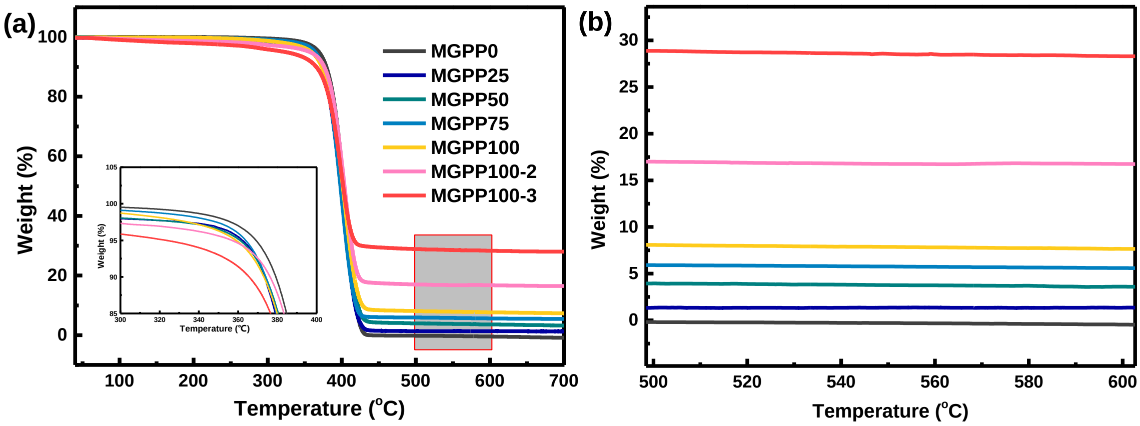

Fig. S10 TGA analysis of MGPP composites

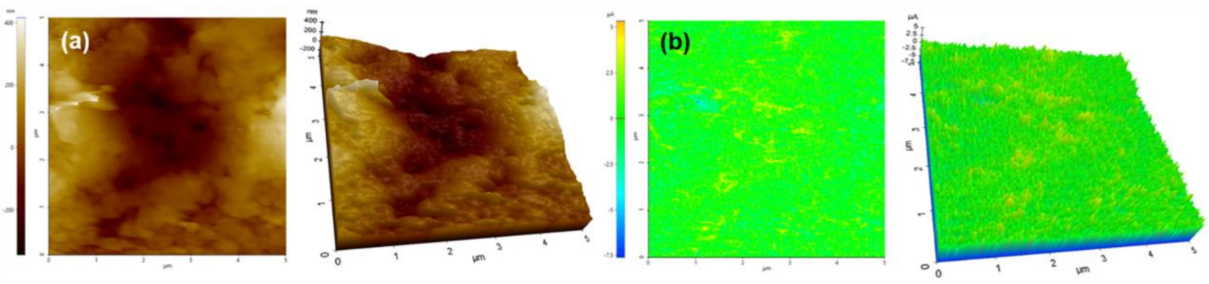

Fig. S11 (**a**) Topography and (**b**) SThM and their corresponding 3D images of MGPP0. The scan size is 5 × 5 μm

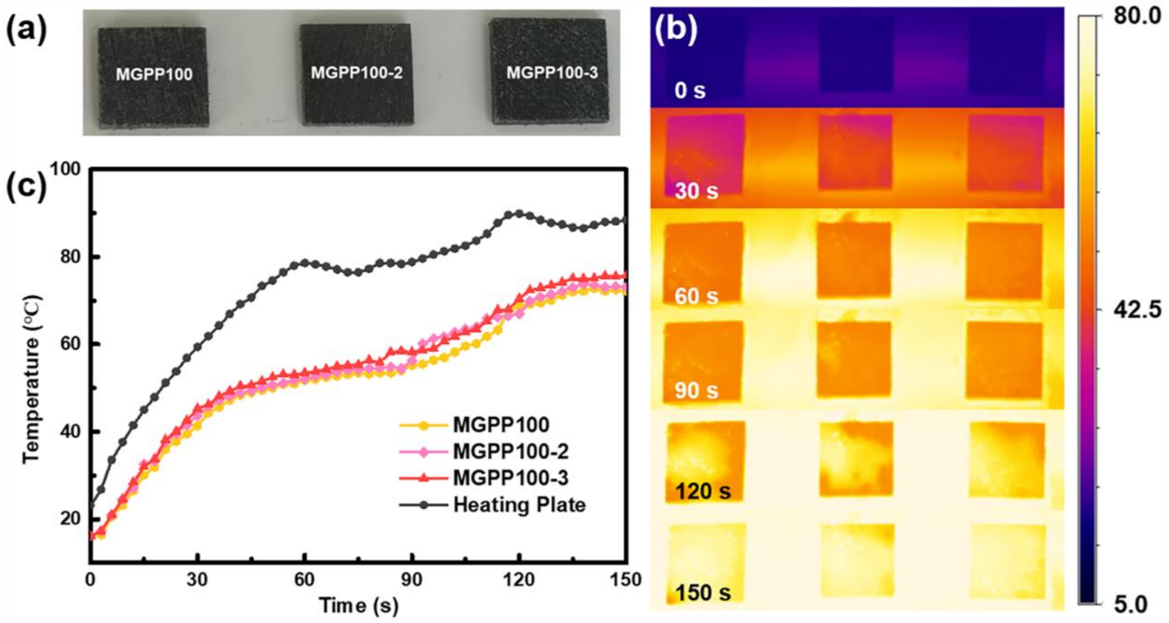

Fig. S12 (**a**) Optical image, (**b**) infrared thermal image, and (**c**) surface temperature variation curve of the thermal conductive MGPP samples

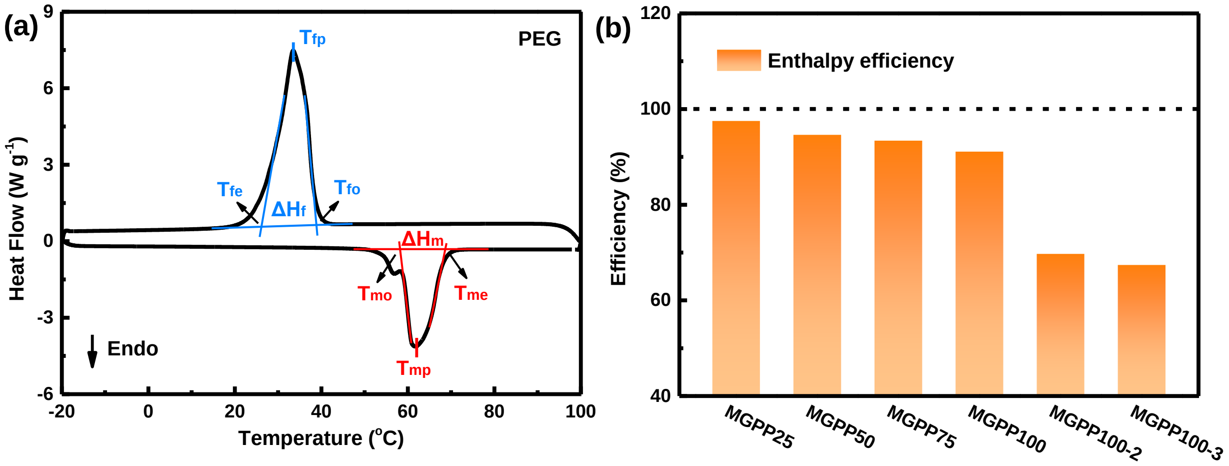

Fig. S13 (**a**) DSC curve of PEG. (**b**) Enthalpy efficiency of MGPP composites

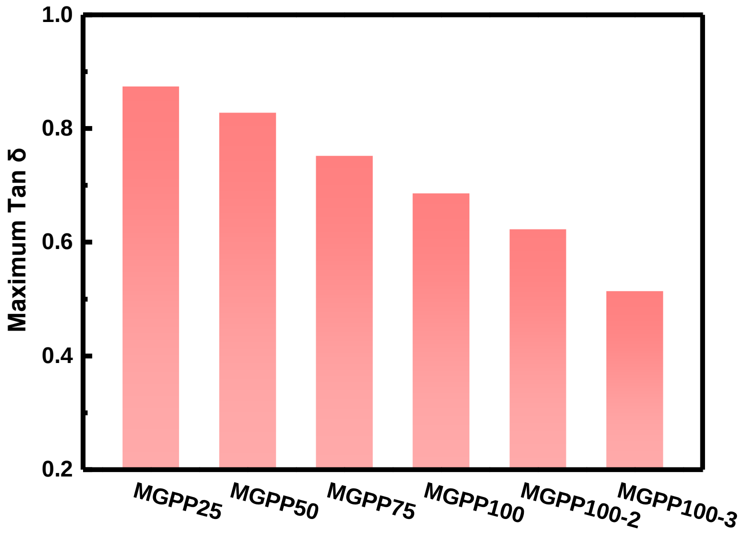

Fig. S14 The maximum tan δ for the MGPP composites

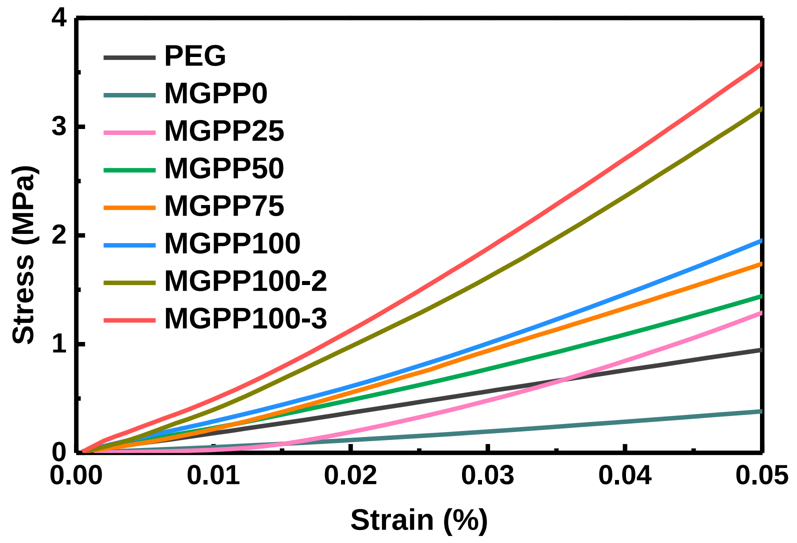

Fig. S15 The enlarged strain-stress curve at the range of 0-0.05% strain

Table S1 DSC data

**Sample**	**T_m (℃)**	**T_c (℃)**	**ΔH_m (J g^{-1})**	**ΔH_c (J g^{-1})**
PEG	61.83	31.06	191.61	192.58
MGPP0	62.07	34.97	189.43	191.25
MGPP25	62.39	36.09	186.94	189.97
MGPP50	62.29	35.44	181.46	188.21
MGPP75	62.41	35.89	179.07	183.56
MGPP100	61.88	36.19	174.72	177.90
MGPP100-2	61.71	37.06	133.74	133.63
MGPP100-3	61.56	37.51	129.32	130.25

Table S2 DSC data

**Samples**	**T_{mo} (℃)**	**T_{me} (℃)**	**T_{co} (℃)**	**T (℃)**
PEG	56.66	65.46	37.38	24.57
MGPP0	58.68	68.11	40.44	24.41
MGPP25	58.12	66.57	39.93	28.67
MGPP50	58.18	66.41	37.09	27.25
MGPP75	58.29	66.69	38.13	27.96
MGPP100	58.32	66.71	39.37	29.53
MGPP100-2	58.31	66.51	38.34	29.29
MGPP100-3	58.04	64.08	38.76	30.85

Where ΔH_m is the melting enthalpy, T_m is the melting temperature, ΔH_c is the crystallization enthalpy, and T_c is the crystallization temperature. The subscript "o" indicates the starting point, "p" indicates the peak point, and "e" indicates the endpoint.

Movie S1 The rapid shape recovery of MGPP100-3 composite spline.

Movie S2 The slow shape recovery of MGPP100-3 composite spline.

Both **Movie S1** and **S2** are processed at 10× speed.
